# Supplementary material for: Decoherence of VB− spin defects in monoisotopic hexagonal boron nitride
Source: Nat Commun. 2022 Jul 27;13:4347. doi: 10.1038/s41467-022-31743-0 (PMC9329290; doi:10.1038/s41467-022-31743-0)
Supplement: Supplementary file 1 — Supplementary Information [file 41467_2022_31743_MOESM1_ESM.pdf]

Supplementary Information: Decoherence of V_B^- spin defects in monoisotopic hexagonal boron nitride

A. Haykal,[1] R. Tanos,[1] N. Minotto,[1] A. Durand,[1] F. Fabre,[1] J. Li,[2] J. H. Edgar,[2]
V. Ivady,[3,4] A. Gali,[5,6] T. Michel,[1] A. Dréau,[1] B. Gil,[1] G. Cassabois,[1] and V. Jacques[1]

[1]*Laboratoire Charles Coulomb, Universit de Montpellier and CNRS, 34095 Montpellier, France*

[2]*Tim Taylor Department of Chemical Engineering,*
Kansas State University, Manhattan, Kansas 66506, USA

[3]*Max Planck Institute for the Physics of Complex Systems,*
Nthnitzer Strae 38, 01187 Dresden, Germany

[4]*Department of Physics, Linkping University, SE-581 83 Linkping, Sweden*

[5]*Wigner Research Centre for Physics,*
P.O. Box 49, H-1525 Budapest, Hungary

[6]*Department of Atomic Physics, Budapest University of Technology and Economics,*
Budafoki ut 8., H-1111 Budapest, Hungary

Supplementary Note 1. Cluster correlation expansion calculations

In order to theoretically study the isotope abundance dependence of the coherence time of the V_B^- center in hBN, we implement the generalized cluster correlation expansion method (gCCE) [1,2], which is the state-of-the-art computational method to describe decoherence of point defect qubits coupled to a bath of nuclear spins. Before applying the method for calculating the coherence time of the V_B^- center, we test our implementation on prevision results published in the literature for the Hahn echo coherence time of the NV center in diamond. As can be seen in Supplementary Figure 2, our simulation reproduces the magnetic field dependence of the coherence time reported in Ref. [3].

In our study, the overall coherence function (C) is obtained as a product of the zero (C_0), first (C_1), second (C_2), and third (C_3) order coherence contributions in gCCE-3 approximation [1,2]. In Supplementary Figure 3, we depict the time dependence of the coherence functions of different orders. As can be seen, the first and second order coherence functions dominate the decay, however, the third order contributions may not be neglected as well.

Nuclear spins and nuclear spin pairs at different distances from the V_B^- center have different effects on the coherence function. Since the dipole-dipole interaction rapidly decreases with the distance of the spins, a proper cut-off radius can be found in general, beyond which the nuclear spins have negligible influence on the coherence properties of the V_B^- center. In our implementation, we define three cut-off radii, see Supplementary Figure 4(a). The first order contribution C_1 accounts only for pair interactions. Convergence of the coherence time with respect to the maximal distance of the electron spin and the nuclear spins is depicted in Supplementary Figure 4(b). As can be seen at $r_1 = 10$ Å the first order contribution converges. The second order coherence contribution C_2 is obtained from three-spin clusters that can account for nuclear spin-nuclear spin flip-flops. Here we define two cut-off radii, r_2 and d_2, that define the maximal distance of the nuclear spins from the V_B^- center and the maximal distance of the nuclear spins in the second order clusters. In Supplementary Figure 4(c,d) we show how the T_2 time converges with respect to these parameters. Convergent results are obtained for $r_2 = 7.5$ Å and $d_2 = 10$ Å. In addition, the first and second neighbor nuclear spins give rise to four body correlation effects in the third order coherence contribution C_3, however, they have only secondary contribution to the decoherence of the V_B^- center in hBN [see Supplementary Figure 3].

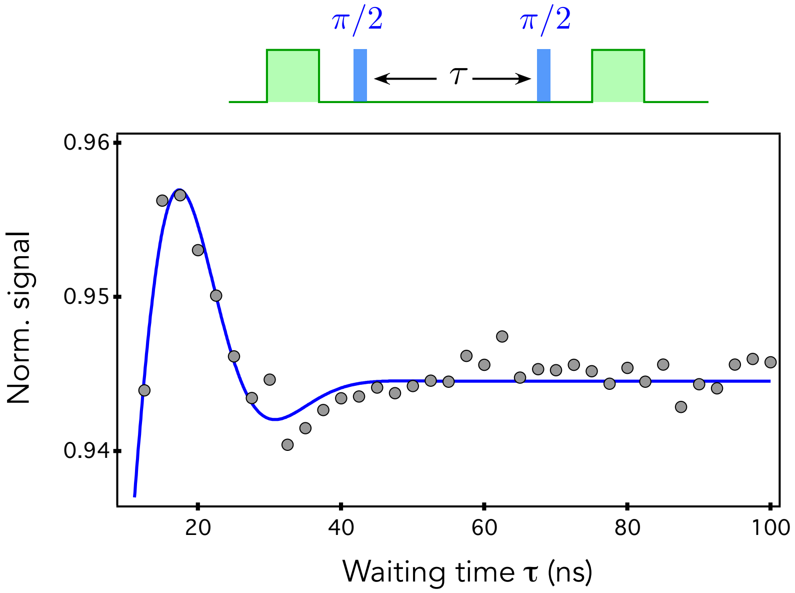

Supplementary Figure 1. Typical free induction decay of an ensemble of V_B^- spin defects hosted in the $h^{10}BN$ crystal, showing a spin dephasing time $T_2^\star \sim 20$ ns. The measurement is carried out under the same experimental conditions as those used to record the data shown in Figure 3 of the main article.

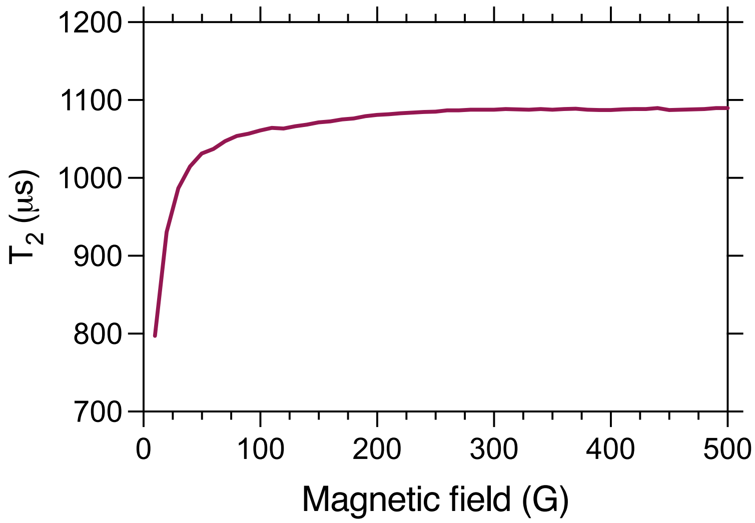

Supplementary Figure 2. Magnetic field dependence of the Hahn echo coherence time of the NV center in diamond.

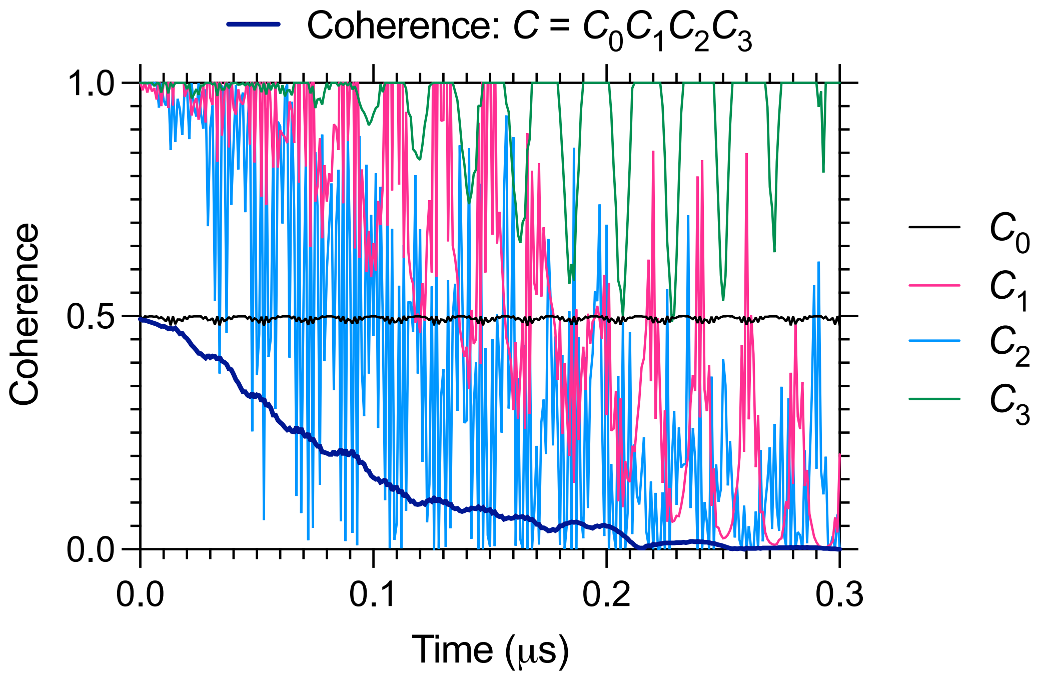

Supplementary Figure 3. Different order contributions to the decay of the coherence function of the V_B^- center in hBN.

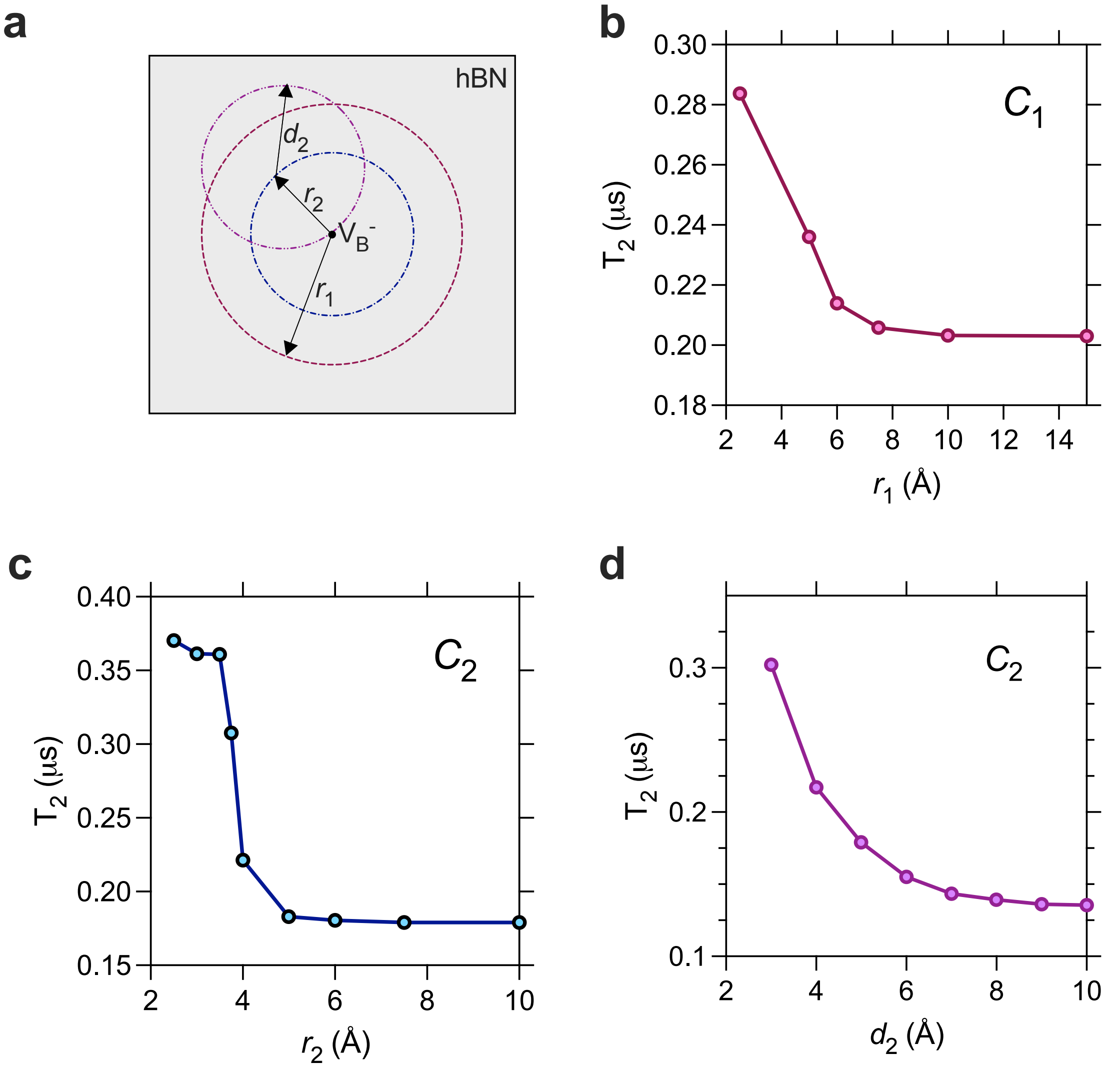

Supplementary Figure 4. Convergence of the coherence time with respect to different cut-off radii of the CCE method. **a** Definition of the different cut off radii. Dependence on **b** the cut off radius r_1 of the first order CCE contribution C_1 and **c** the cut off radius r_2 of the second order CCE contribution C_2. **d** Convergence of the coherence time with respect to the maximal distance d_2 of the nuclear spins in the second order contribution.

Supplementary References

[1] W. Yang and R.-B. Liu, Physical Review B 78, 085315 (2008).

[2] M. Onizhuk, K. C. Miao, J. P. Blanton, H. Ma, C. P. Anderson, A. Bourassa, D. D. Awschalom, and G. Galli, PRX Quantum 2, 010311 (2021).

[3] H. Seo, A. L. Falk, P. V. Klimov, K. C. Miao, G. Galli, and D. D. Awschalom, Nature Communications 7, 12935 (2016).
